# Update on Conventional Osteosarcoma

**DOI:** 10.1055/s-0043-1771483

**Published:** 2024-01-29

**Authors:** Luiz Eduardo Moreira Teixeira, Alex Guedes, Suely Akiko Nakagawa, Karine Corrêa Fonseca, Eduardo Ribeiro Lima

**Affiliations:** 1 Departamento do Aparelho Locomotor, Faculdade de Medicina, Universidade Federal de Minas Gerais, Belo Horizonte, MG, Brasil; 2 Grupo de Oncologia Ortopédica, Hospital Santa Izabel, Santa Casa de Misericórdia da Bahia, Salvador, BA, Brasil; 3 Centro de Sarcomas e Tumores Ósseos, AC Camargo Cancer Center, São Paulo, SP, Brasil; 4 Grupo de Oncologia Pediátrico, Hospital das Clínicas, Belo Horizonte, MG, Brasil

**Keywords:** neoplasms, ostesarcoma, sarcoma

## Abstract

Conventional osteosarcoma is a high-grade malignant tumor characterized by the production of osteoid matrix by malignant osteoblasts. It typically affects the long bones of children and adolescents. Treatment includes systemic chemotherapy and a local surgical approach with wide resection. Recent advances in oncology concepts, imaging, surgical planning, and cancer treatment protocols allow for improved survival and a higher limb preservation rate. This paper addresses the current status regarding the incidence, pathology, treatment, and prognosis of conventional high-grade osteosarcoma.

## Introduction

Bone sarcomas are relatively rare tumors with a North American and European incidence of 0.75 to 2.0 cases per 100,000 people. 1 Osteosarcoma is the most common bone sarcoma. 1 2 3 Even though its rarity limits demographic studies, the age distribution is bimodal, 1 2 with a first peak in the second decade of life and a second, smaller peak in older adults (30% of osteosarcoma cases occur in individuals > 40 years) and related to secondary tumors (post-irradiation, Paget disease).

A study recently described the incidence and survival rates of 5,016 patients with osteosarcoma followed in the United States between 1975 and 2017 using the Surveillance, Epidemiology, and End Results (SEER) Program. 3 The study analyzed patients per age, race/ethnicity, histologic subtype, stage, and tumor location. The authors observed a similar incidence of primary osteosarcoma between genders and a steadily increasing relative 5-year survival rate in children aged 0 to 9. In Afro-Americans, the highest incidence occurred among older people; this incidence increased significantly over the study period. Overall, survival rates have remained relatively unchanged over recent decades; it is lower in older patients and those presenting metastatic disease, axial skeleton tumors, and subsequent relapse. In patients aged 0 to 24, the incidence of subsequent osteosarcoma relapses has tripled since the 2000s.

There are few epidemiological studies on osteosarcoma in Brazil.

An epidemiological, retrospective study of 184 cases 4 treated between 1974 and 1994 showed a slight preference for males (1.3/1.0), with an age range from 6 to 78 years old (peak incidence, 10 to 19 years old [63.6% of cases, n = 117]), an overall mean age of 18.7 years old, and no evidence of a second peak in older subjects. The incidence was 68.5% (n = 126) in blacks and browns and 31.5% (n = 58) in whites, with a 2.2 ratio. Most cases originated in long bones (97.3%, n = 179), especially the distal segments of the femur and proximal portions of the tibia.

A study with patients treated between 1991 and 2002 at a single Pediatric Oncology service in São Paulo, SP, Brazil, evaluated 60 osteosarcoma cases in patients aged 5 to 16 (median age, 11 years old). Seventy percent of the patients were older than 10; 61.7% were male, and 65% were non-white. All patients presented involvement of the appendicular skeleton (51.7% in the distal segment of the femur, 23.2% in the proximal region of the tibia, 10.0% in the proximal humerus, 6.7% in the proximal femur, 5.0% in the middle segment of the femur, 1.7% in the distal portion of the tibia, and 1.7% in the distal fibula). A fourth of these patients had pulmonary metastasis at diagnosis, including three subjects with concomitant metastases elsewhere. Regarding the histological subtype, 53.3% of the osteosarcomas were osteoblastic, 20.0% were chondroblastic, 8.3% were fibroblastic, 5.0% were telangiectatic, 1.7% were small cells, and 11.7% were indeterminate. 5

A retrospective study with osteosarcoma patients treated in Teresina, PI, Brazil, from 2005 to 2010 identified 32 subjects aged 6 to 73 (median age, 15 years old), predominantly male, black, and presenting conventional osteosarcoma of the osteoblastic type (71.8 %) at an appendicular location. 6

Another Brazilian study analyzed the epidemiological characteristics of adolescents (10 to 19 years old) with neoplasms treated between 2000 and 2006 at the Institute of Pediatric Oncology of the Federal University of São Paulo. From 2,362 patients admitted in this period with a cancer diagnosis, 14.6% had osteosarcoma, second in frequency in this casuistry. 7

## Pathology

The World Health Organization revised and published the histological classification of osteosarcomas in 2020. 2 The most prevalent histological subtype is conventional or classic osteosarcoma. Other subtypes are less aggressive, such as low-grade central and parosteal osteosarcoma. Periosteal osteosarcoma shows an intermediate grade, while conventional primary, secondary, and high-grade superficial osteosarcomas are aggressive. This paper focuses on conventional or classic osteosarcoma.

### Conventional Osteosarcoma

**Fig. 1:**
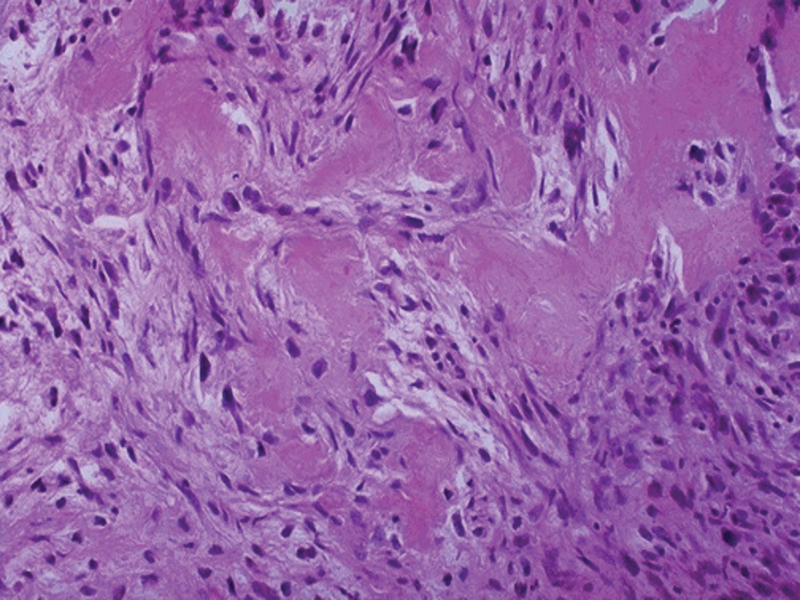
Malignant osteoblasts producing osteoid matrix. Source: Author's personal file.

Conventional osteosarcoma is a high-grade intramedullary sarcoma in which tumor cells produce bone ( Fig. 1 ). It has three subtypes: conventional, telangiectatic, and small cell. Conventional osteosarcoma commonly contains varying amounts of cartilaginous and/or fibroblastic neoplastic components. Per the predominant matrix, this subtype is classified as osteoblastic (76-80%), chondroblastic (10-13%), or fibroblastic (10%). 2

Conventional osteosarcoma can affect any bone, but most arise in the metaphysis of long bones (90%), often in the distal segment of the femur (30%), followed by the proximal tibia (15%) and proximal humerus (15%). These sites concentrate the most active epiphyseal plates in the growing skeleton. In addition, conventional osteosarcomas are infrequent in the diaphysis (9%) and rare in the epiphysis. 8 9 10 11 12 13 The annual incidence of conventional osteosarcoma is 4.4 cases per million in the age group from 0 to 24 years, 1.7 cases per million from 25 to 59 years old, and 4.2 cases per million people older than 60. The distribution of these neoplasms shows a predominance for males (1.3:1).

**Fig. 2:**
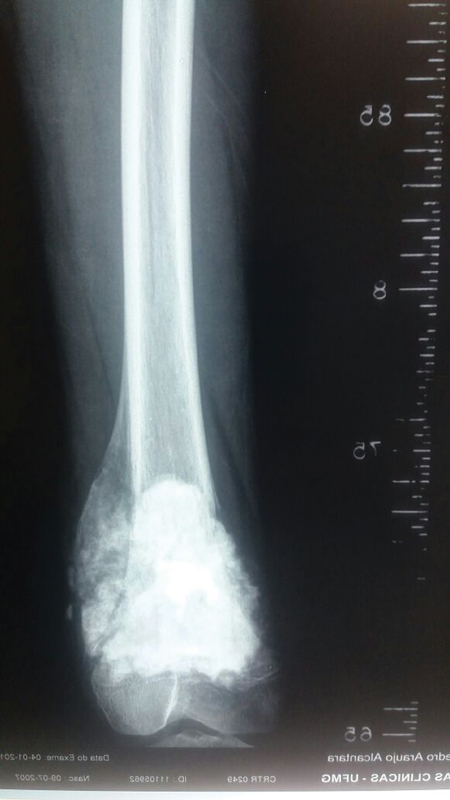
Radiographic appearance of conventional osteosarcoma. Source: Author's personal file.

Conventional osteosarcoma causes permeative bone destruction and mineralization of the tumor matrix, giving a mixed (lytic/sclerotic) appearance with poorly defined, cotton-wooled, immature tumor ossification. Non-expansive bone destruction and periosteal detachment are frequent, resulting in reactive new bone, typically oriented perpendicular to the tumor but also parallel (“onion skin”) or divergent (“sunbeams”). Periosteal detachment can be interrupted in the central portion of the lesion, leading to the formation of a Codman's triangle. An extraosseous extension is common, often eccentric and mineralized ( Fig. 2 ). 8 9 10 11 Bone scanning reveals increased uptake at the lesion site, corresponding to the osteoblastic areas of the tumor. Magnetic resonance imaging (MRI) shows heterogeneous intermediate signals on T1-weighted images and hyperintensity on fluid-sensitive sequences, in addition to hyperintense hemorrhagic areas and hypointense mineralized areas. Periosteal ossification may form low-signal radiating wires; the outer portion of the periosteum may form a capsule, often presenting with focal rupture. 10 The viable tumor presents enhancement after contrast agent administration. Calcified areas remain hypointense, and chondroblastic areas may show nodular septal enhancement.

## Treatment

### Surgical Treatment – Osteosarcoma

The standard surgical treatment of conventional osteosarcoma is wide resection since studies correlate positive margins with reduced survival. 12 13 14 15 16 The normal tissue around the tumor must present 0.5 and 2 cm in thickness. However, these values remain controversial. The longitudinal bone margin must be 3 cm and have a layer of soft tissue around the tumor. Nevertheless, recent literature does not demonstrate a worse oncological outcome in resections with 1.5 cm margins. 17 18

He et al. 15 reemphasized the basic principle of a specific margin in a 2016 meta-analysis and the role of negative margins in reducing local recurrence from osteosarcomas of the extremities and pelvic region. 15 16

Bone marrow margin assessment with frozen sections can confirm negative margins or guide further resections. However, later final pathological analyzes are essential to corroborate the margins, determine the response to neoadjuvant chemotherapy (percentage of necrosis), and disclose other relevant information. 16

Immediately after osteotomy, tamponade of the medulla close to the tumor with bone wax prevents tumor extravasation and dissemination of neoplastic cells in the surgical bed.

More than 85% of patients with osteosarcoma in the extremities may undergo limb-sparing surgeries. Indications for these procedures include the potential achievement of local control and proper limb function. Amputation is indicated when limbs are not functional and free oncological margins are not feasible.

Preoperative planning must consider neurovascular involvement, the presence of pathological fractures, the remaining potential for skeletal growth, and the patient's postoperative expectations.

Although associated with greater psychosocial satisfaction and a higher Musculoskeletal Tumor Society Staging System (MSTS) score, limb-sparing surgeries have more complications and require more revision procedures. 12

There is no prospective, randomized study comparing patients undergoing amputation or limb-sparing surgery. However, some retrospective studies show no statistical difference in the overall survival of patients treated with amputation versus limb preservation. 12

Fractures and their relationship with local recurrence are controversial. Ferguson et al. showed that a pathological fracture did not prevent limb-sparing surgery or increase local recurrence; however, these patients had worse overall survival. 14

If the fracture occurs before or during neoadjuvant treatment, there is usually an attempt to achieve stabilization with simple immobilization until the end of neoadjuvant therapy. This is followed by restaging for surgical planning.

Limb-sparing surgery is often indicated when resection with local control and adequate function is achievable. Once sparing is possible, the surgery has three steps: resection with proper margins, reconstruction, and reconstruction coverage. Reconstruction must be long-lasting and restore limb function. 19

There is no consensus on the ideal sequence for assessment during surgical planning. It has been suggested to consider T1-weighted MRI with no fat suppression before chemotherapy. 13 16

Many tumors destroy the cortex, invade soft tissues, and grow towards and adjacent to vascular and nervous bundles, challenging tumor resection with free margins. Historically, amputation levels were high; this changed in 1990 when the number of limb-sparing surgeries increased. However, this increase did not result in survival gain. 19

Options for reconstruction include endoprosthesis, graft and prosthesis composition, free or vascularized graft, arthrodesis, gyroplasty, or amputation. The surgical method will depend on the tumor, the patient's age, the functional capacity of the limb, and the prognosis.

Surgical navigation helps to increase the precision of osteotomies for pelvic and periarticular osteosarcomas. Planning uses MRI and computed tomography (CT) and may require creating a 3D model for the procedure, guides, or wedges to assist osteotomies or customized endoprostheses. 13 However, the current limitation is the lack of navigation allowing real-time visualization and any required adjustments. 13

**Fig. 3:**
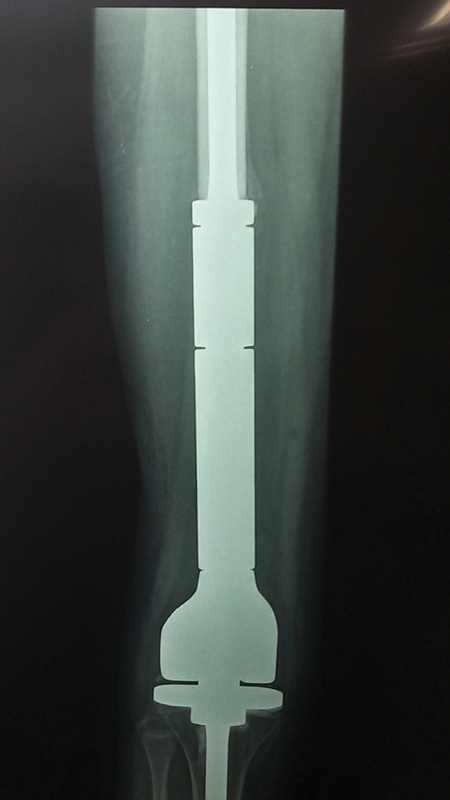
Reconstruction with non-conventional distal femur endoprosthesis. Source: Author's personal file.

Reconstructions with endoprostheses present satisfactory functional outcomes and early return of weight bearing. Even though several options are available, implant-related complications, aseptic loosening, and infection remain challenges that advanced technology tries to overcome ( Fig. 3 ).

Allografts can be used milled, structurally, or both depending on the required reconstruction. The most common complications include pseudarthrosis, graft fracture, and infection.

Aponte-Tiao et al. 20 evaluated 193 patients with segmental allografts for 10 years and identified high rates of pseudarthrosis and fracture, in addition to a 14% infection rate. In the end, allograft removal occurred in 40% of subjects, who underwent prosthesis placement or amputation.

Autografts include free or vascularized grafts. There is also the possibility of using bone autograft after tumor devitalization by different methods, either heat, cooling, or radiation. 13

Current cryotherapy techniques with liquid nitrogen can be performed either with a free segment or a pedicled graft, maintaining the vascular bundle. A comparative study showed that the pedicled graft consolidates faster. 21

## Systemic Treatment

Before the advent of chemotherapy, the only treatment for conventional osteosarcoma was surgery. Despite the early amputation strategy proposed by Gross, the overall 5-year survival remained low, at 20%, 22 23 24 and lung metastases were observed after 6 to 12 months, suggesting the presence of micrometastases at diagnosis.

The introduction of multidrug chemotherapy reduced the systemic spread of the disease and improved the 2-year overall survival by 50%, as demonstrated by Eiber et al. 25 In 1976, Rosen et al. 26 introduced neoadjuvant chemotherapy followed by conservative surgery, which proved safe and allowed the analysis of surgical margins and histological response to treatment. However, survival has not increased over the past 30 years, despite the constant search for new therapies.

Today, treatment consists of neoadjuvant multidrug therapy, wide resection, and adjuvant multidrug therapy. The main chemotherapy drugs include high-dose methotrexate, cisplatin, and doxorubicin. 27 Ifosfamide and etoposide are additional active agents often used in high-risk and/or relapsed patients.

Switching drugs based on histologic response to neoadjuvant chemotherapy was assessed and failed to demonstrate a survival improvement. The primary objectives of the European and American Osteosarcoma Study Group (EURAMOS-1) clinical trial were to determine whether the addition of postoperative ifosfamide and etoposide would improve outcomes in cases of poor response to neoadjuvant chemotherapy and whether the addition of 2-year maintenance treatment with alpha-2 interferon would improve outcomes for good responders. Unfortunately, there was no significant difference between the study arms. 27

The disease recurs in approximately 30% of non-metastatic patients, especially in the lungs. Salvage therapy for these patients remains poor, with a 5-year survival expectancy of only 25%. 28 A treatment regimen using drugs such as gemcitabine and docetaxel is an option but with low response. The association of temozolamide and etoposide has been evaluated and shown potential benefits as rescue therapy. 29

Long-term metronomic chemotherapy in low doses acts on endothelial cells of the neovessels formed in the tumor microenvironment, inhibiting local angiogenesis. This method showed little encouraging results in non-metastatic patients. 30 For metastatic patients, oral cyclophosphamide, methotrexate, and etoposide provide better outcomes. Metastatic disease at diagnosis, relapse, and multiple-drug resistance (MDR) are the three major obstacles to a good clinical course and better chances of cure.

Osteosarcoma is a relatively radioresistant tumor. Today, only unresectable tumors and advanced diseases undergo radiotherapy, especially for pain control.

Recent advances include inhaled chemotherapy, which directly exposes the lung, a significant metastasis site, to high drug doses, with low systemic toxicity. 31 In the future, this treatment modality may allow administering the administration of immunotherapies such as interleukin 2 (IL-2). 32 The literature has some related preclinical studies, particularly an open, phase I/II study using inhaled gemcitabine from Gordon et al. 33 that may be the first evidence of the clinical efficacy of this treatment.

Histological and immunohistochemical assessment of the primary tumor can produce useful prognostic markers and help plan a more individualized treatment. Microscopic vascular invasion, observed in some tumor specimens, may be associated with a poor response to neoadjuvant chemotherapy, a high risk of metastases, and shorter survival. This observation suggests that, in these cases, one could evaluate the achievement of a wider tumor margin and discuss using adjuvant treatment schemes with alternative agents.

Osteosarcoma is highly heterogeneous, both in histological analysis and molecular classification. Recently, several state-of-the-art sequencing studies have revealed significant signaling pathways in osteosarcoma. Targeted therapy is likely most successful when the matching target is clear. Studies tried to classify osteosarcomas based on frequent, validated genetic alterations. A review article published in 2021 by Chen et al. 34 classified osteosarcoma into ten molecular subtypes, including osteosarcomas with cyclin-dependent kinases/CDK, MYC, MDM2, AURKB, and RTK amplification; RTK amplification; P13K/AKT change; NF, BRCA, and ATRX deletions, and IGF mutation. Drugs from the mTOR, MEK, EGF-1R, VEGFR, and PARP inhibitor families are under study in preclinical trials.

In recent years, numerous clinical trials have been conducted with immunotherapies and have received considerable attention for their effectiveness in treating various tumors. Preclinical trials of immunotherapies involving tumor vaccines, immunomodulators, genetically modified T cells, cytokines, immunological checkpoint inhibitors, or combined treatments are under study to treat osteosarcoma. A better understanding of the immune response to osteosarcoma and the development of biomarkers could increase the number of patients who will benefit from immunotherapies. 35

CAR-T cell therapy has been intensively studied in the last few years, especially for treating hematologic malignancies. Studies using it to treat solid tumors are ongoing. Although access remains difficult, CAR-T cell therapy can be promising. An open clinical trial investigates the treatment of positive anti-GD2 sarcomas and neuroblastomas. 36 37

Currently, the overall 5-year survival is around 65% in studies from the United States. However, survival rates have remained at a plateau since the 1970s. Factors for poor prognosis include metastasis at diagnosis, female gender, increased alkaline phosphatase level, secondary tumors, expression of microRNAs, and Huvos I and II index ( Table 1 )

**Table 1:** Tumor necrosis index and response to neoadjuvant chemotherapy

RESPONSE	HUVOS INDEX	NECROSIS
GOOD RESPONDER	IV	100%
GOOD RESPONDER	III	>90%
BAD RESPONDER	II	50% TO 90%
BAD RESPONDER	I	UP TO 50%

### Final Considerations

Advances in systemic and local control therapies continue to evolve. A better knowledge of the biology and dissemination mechanisms is essential for a more effective therapeutic approach, especially for patients with poor prognosis. The near future brings patient stratification based on genetic characteristics and the development of targeted therapies.
