# Supplementary material for: Update on Conventional Osteosarcoma
Source: Rev Bras Ortop (Sao Paulo). 2024 Jan 29;59(6):e815–20. doi: 10.1055/s-0043-1771483 (PMC11663062; doi:10.1055/s-0043-1771483)
Supplement: Related texts

# Research article: Atualização sobre osteossarcoma convencional

**Keywords:** neoplasias, osteossarcoma, sarcomas

## Abstract

O osteossarcoma convencional é um tumor de alto grau de malignidade caracterizado pela produção de matriz osteóide pelos osteoblastos malignos. Tipicamente acomete ossos longos de crianças e adolescentes e o tratamento inclui abordagem sistêmica com quimioterapia e abordagem cirúrgica local com ressecção ampla. Avanços recentes nos conceitos oncológicos, de imagem, planejamento cirúrgico e protocolos de tratamento oncológico permitem uma melhora na sobrevida e maior taxa de preservação de membros. Este artigo aborda o estado atual relativo a incidência, patologia, tratamento e prognóstico do osteossarcoma convencional de alto grau.

## Introdução

Os sarcomas ósseos são tumores relativamente raros com incidência na América do Norte e Europa de 0,75 a 2,0 casos por 100.000 habitantes. 1 Dentre esses, o osteossarcoma é o mais comum. 1 2 3 Embora sua raridade limite estudos demográficos, observa-se uma distribuição etária bimodal 1 2 com primeiro pico na segunda década de vida e um segundo pico, de menor monta, em adultos mais velhos (30% dos casos de osteossarcoma ocorrem em indivíduos > 40 anos) relacionado a tumores secundários (pós-irradiação, doença de Paget).

Estudo recentemente descreveu a incidência e taxas de sobrevida de 5016 pacientes portadores de osteossarcoma acompanhados nos Estados Unidos da América entre os anos de 1975 e 2017, utilizando o programa *Surveillance, Epidemiology, and End Results* (SEER). 3 Os pacientes foram analisados por idade, raça/etnia, subtipo histológico, estágio, e localização do tumor. Os autores observaram que, na faixa etária de 0 a 9 anos, a incidência de osteossarcoma primário foi semelhante entre os sexos, e a sobrevida relativa de cinco anos aumentou de forma constante ao longo do tempo. Os pretos apresentaram a maior incidência entre os idosos, com aumento significativo da incidência ao longo do período de estudo. No geral, as taxas de sobrevida para todos os casos permaneceram relativamente inalteradas nas últimas décadas, com pior sobrevida observada em pacientes mais velhos, doença metastática, tumores situados no esqueleto axial, e recidiva subsequente. Nos pacientes de 0 a 24 anos, a incidência de recidivas subsequentes de osteossarcoma triplicou desde a década de 2000.

No Brasil, há poucos estudos epidemiológicos versando sobre o osteossarcoma.

Estudo retrospectivo epidemiológico de 184 casos 4 atendidos entre 1974 e 1994 apresentou discreta predileção pelo sexo masculino (1,3/1,0); idade entre seis e 78 anos - pico de incidência entre dez e 19 anos (63,6% dos casos, n = 117), com idade média global de 18,7 anos e sem evidência de segundo pico em indivíduos mais velhos. A média de idade global foi de 18,7 anos. A incidência em pretos e pardos foi de 68,5% (n = 126) e em brancos, de 31,5% (n = 58), com proporção de 2,2. A grande maioria dos casos originou-se nos ossos longos (97,3%, n = 179) nos segmentos distal do fêmur e proximal da tíbia.

Estudo realizado com pacientes atendidos entre 1991 e 2002 em um único serviço de Oncologia Pediátrica de São Paulo, SP, avaliou 60 casos de osteossarcoma em pacientes com idade entre cinco e 16 anos (mediana de 11 anos), sendo 70% com idade igual ou superior a 10 anos; 61,7% eram do sexo masculino e 65% eram de etnias não brancas. Todos os pacientes apresentavam comprometimento do esqueleto apendicular (51,7% no segmento distal do fêmur, 23,2% na região proximal da tíbia, 10,0% no úmero proximal, 6,7% no fêmur proximal, 5,0% no segmento médio do fêmur, 1,7% no segmento distal da tíbia e 1,7% na fíbula distal). Um quarto dos pacientes apresentava metástase pulmonar ao diagnóstico; destes, três apresentavam metástases concomitantes em outros locais. Com relação ao subtipo histológico, observaram 53,3% dos osteossarcomas eram osteoblásticos; 20,0% condroblásticos; 8,3% fibroblásticos; 5,0% telangiectásicos; 1,7% de pequenas células; e, em 11,7%, o subtipo não foi determinado. 5

Estudo retrospectivo envolvendo portadores de osteossarcoma atendidos em Teresina-PI entre 2005 e 2010 identificou 32 pacientes com faixa etária situada entre seis e 73 anos (mediana de 15 anos), predomínio do sexo masculino, pretos e osteossarcoma convencional do tipo osteoblástico (71,8%), de localização apendicular. 6

Em outro estudo nacional foram analisadas as características epidemiológicas de adolescentes (10−19 anos) portadores de neoplasias atendidos entre os anos de 2000 e 2006 no Instituto de Oncologia Pediátrica da Universidade Federal de São Paulo. Do total de 2.362 pacientes admitidos neste período com diagnóstico de câncer, 14,6% eram portadores de osteossarcoma, segundo colocado em frequência nesta casuística. 7

## Patologia

A classificação histológica dos osteossarcomas foi revista e publicada pela Organização Mundial da Saúde em 2020. 2 O subtipo histológico mais prevalente é o osteossarcoma convencional ou clássico. Outros subtipos são considerados menos agressivos como central de baixo grau e o parosteal; o osteossarcoma periosteal é considerado de grau intermediário, enquanto o osteossarcoma convencional primário, secundário, e superficial de alto grau, são consideradas de agressivos. Este artigo irá abordar seu conteúdo baseado no osteossarcoma convencional ou clássico.

## Osteossarcoma Convencional

Osteossarcoma convencional é um sarcoma intramedular de alto grau no qual as células tumorais produzem osso ( Fig. 1 ). Apresenta três subtipos: convencional, telangiectásico, e de pequenas células. O osteossarcoma convencional comumente costuma conter quantidades variadas de componentes neoplásicos cartilaginosos e/ou fibroblásticos – tomando como base a matriz predominante, este subtipo pode ser classificado como osteoblástico (76-80%), condroblástico (10-13%) ou fibroblástico (10%). 2

**Fig. 1:** Osteoblastos malignos produzindo matriz osteóide. Fonte Arquivo pessoal do autor.

O osteossarcoma convencional pode afetar qualquer osso, mas a maioria surge na metáfise dos ossos longos (90%), frequentemente no segmento distal do fêmur (30%), seguido pelo segmento proximal da tíbia (15%) e proximal do úmero (15%), sítios que concentram as placas epifisárias mais ativas no esqueleto em crescimento, são infrequentes na diáfise (9%), e, raros, na epífise. 8 9 10 11 12 13 O osteossarcoma convencional apresenta incidência anual de 4,4 casos por milhão na faixa de idade situada entre 0 e 24 anos; 1,7 casos por milhão entre 25 e 59 anos; e 4,2 casos por milhão de habitantes > 60 anos. Há predominância do gênero masculino (1,3:1) na distribuição destas neoplasias.

O osteossarcoma convencional apresenta destruição óssea permeativa, associada à mineralização da matriz tumoral, dando aparência mista (lítica/esclerótica), com ossificação tumoral imatura mal definida, algodonosa. Frequentemente, há destruição óssea não expansiva e descolamento do periósteo, que produz osso novo reativo, tipicamente orientado de maneira perpendicular ao tumor, podendo apresentar arranjo paralelo (“casca de cebola”) ou divergente (“raios de sol”). O descolamento periosteal pode ser interrompido na porção central da lesão e resultar na formação do triângulo de Codman. Extensão extraóssea é comum e frequentemente excêntrica e mineralizada ( Fig. 2 ). 8 9 10 11 O mapeamento ósseo apresenta captação aumentada no sítio da lesão, correspondendo às áreas osteoblásticas do tumor. Na ressonância magnética (RM) apresenta sinal intermediário heterogêneo nas imagens ponderadas em T1 e hiperintensidade nas sequencias sensíveis a fluidos, com áreas hemorrágicas hiperintensas e áreas mineralizadas hipointensas - a ossificação periosteal pode ser observada sob a forma de fios radiantes de baixo sinal; a porção externa do periósteo pode formar uma cápsula que frequentemente apresenta-se ruptura focal. 10 Após a administração do contraste, o tumor viável realça, as áreas calcificadas permanecem hipointensas, e as áreas condroblásticas podem apresentar realce septal nodular.

**Fig. 2:** Aspecto radiográfico do osteosarcoma convencional. Fonte arquivo pessoal do autor.

## Tratamento

## Tratamento Cirúrgico - Osteossarcoma

O tratamento cirúrgico padrão do osteossarcoma convencional é a ressecção ampla, uma vez que estudos correlacionam margens positivas com queda da sobrevida. 12 13 14 15 16 A espessura do tecido normal ao redor do tumor deva ter entre 0,5 e 2 cm, embora ainda seja controverso e a margem óssea longitudinal seja de 3 cm com uma camada de tecido de partes moles ao redor do tumor, embora a literatura recente não tenha demonstrado piora no resultado oncológico nas ressecções com margens de 1,5 cm. 17 18

He et al. 15 reenfatizaram o princípio básico sobre a margem específica em uma metanálise de 2016, e sua relação em reduzir a recorrência local com margens negativas nos casos de osteossarcomas de extremidades e região pélvica. 15 16

A avaliação da margem óssea medular com exames de congelação pode ser realizada para confirmar margens negativas ou guiar ressecções adicionais, entretanto as análises patológicas finais realizadas posteriormente são essenciais para confirmar as margens, resposta à quimioterapia neoadjuvante (percentual de necrose) e outras informações relevantes. 16

Imediatamente após a osteotomia, o tamponamento da medular junto ao tumor com cera de osso, evita o extravasamento tumoral e disseminação de células neoplásicas no leito cirúrgico.

Cirurgias preservadoras de membro são possíveis em mais de 85% dos pacientes com osteossarcoma em extremidades, e estão indicadas quando o controle local pode ser obtido, em conjunto com uma função adequada do membro. Quando essa função e as margens oncológicas livres não puderem ser obtidas, a amputação deve ser indicada.

Durante o planejamento pré-operatório, deve-se considerar o envolvimento neurovascular, presença de fratura patológica, potencial remanescente de crescimento do esqueleto e as expectativas pós-operatórias do paciente.

As cirurgias preservadoras, embora estejam associadas com maior satisfação psicossocial e escore mais elevado de MSTS apresentam mais complicações e maior número de cirurgias de revisões. 12

Não existe trabalho prospectivo e randomizado que compare pacientes com amputação e paciente submetidos a preservação de membros, mas alguns estudos retrospectivos não evidenciam diferença estatística na sobrevida global dos pacientes tratados com amputação versus preservação de membro. 12

Existe controvérsia nos casos de fraturas, e sua relação com recorrência local. Ferguson e cols. mostraram que a presença da fratura patológica não impediu a cirurgia preservadora de membro, e não houve aumento na recidiva local, mas apresentaram pior sobrevida global. 14

Usualmente, se a fratura ocorrer antes ou durante o tratamento neoadjuvante, tenta-se obter uma estabilização com uma imobilização simples até o término do tratamento neoadjuvante. E então, o reestadiamento é realizado para o planejamento cirúrgico.

A cirurgia preservadora de membro normalmente é indicada quando a ressecção com controle local e função adequada são possíveis de serem obtidas. Uma vez possível a preservação a cirurgia tem três passos ressecção com margens adequadas, reconstrução e cobertura da reconstrução. A reconstrução deve ser duradoura e que restaure a função do membro. 19

Não existe consenso sobre a sequência ideal para avaliação no planejamento cirúrgico. Sugere considerar a RM em T1 sem supressão de gordura realizadas pré-quimioterapia. 13 16

Muitos tumores destroem a cortical, invadem as partes moles e crescem em direção e adjacente ao feixe vasculo-nervoso, e a ressecção tumoral com margens livres, torna-se desafiador. Historicamente, os níveis de amputações eram elevados; e desde 1990 tem ocorrido uma mudança, com um maior número de cirurgias preservadoras de membros, entretanto, não tem sido relacionada com ganho de sobrevida. 19

As opções de reconstruções podem ser com endoprótese, composição de enxerto e prótese, enxerto livre ou vascularizado, artrodese, giroplastia ou amputação. O método cirúrgico dependerá do tumor, da idade do paciente, da capacidade funcional do membro e do prognóstico.

Cirurgias navegadas podem ser úteis para alcançar maior precisão nas osteotomias dos osteossarcomas pélvicos e periarticulares. O planejamento é feito com RM e TC, e pode ser necessário a elaboração de um modelo 3D para o procedimento, criando guias ou cunhas que auxiliem nas osteotomias ou para a elaboração de endopróteses customizadas. 13 Entretanto, a limitação atual é a falta de serras de navegação que permitam a visualização e ajustes necessários em tempo real. 13

As reconstruções com endopróteses apresentam resultados funcionais satisfatórios e retorno precoce de descarga de peso, e por isso são opções de escolha em muitos casos, apesar das complicações relacionadas ao implante, soltura asséptica e infecção que continuem sendo desafios que os avanços da tecnologia tentam suplantar ( Fig. 3 ).

**Fig. 3:** Reconstrução com endoprótese não convencional de femur distal. Fonte arquivo pessoal do autor.

Os aloenxertos podem ser utilizados moídos e/ou estruturais, a depender do tipo de reconstrução necessária. As complicações mais comuns são pseudoartrose, fratura do enxerto e infecção.

Um estudo de Aponte-Tiao et al. 20 avaliaram 193 pacientes com aloenxertos segmentares por um período de 10 anos, e identificaram elevadas taxas de pseudoartrose e fratura; e taxas de14% de infecção. Ao final, houve 40% de retirada do aloenxerto, com colocação de prótese ou amputação.

O autoenxerto pode ser de enxerto livre ou vascularizado. Existe também a possibilidade do uso do autoenxerto ósseo após a desvitalização do tumor por diferentes métodos, seja por calor, esfriamento ou radiação. 13

As técnicas atuais de crioterapia com nitrogênio líquido, podem ser realizadas tanto com o segmento livre quanto com enxerto pediculado, mantendo-se o feixe vascular. Um estudo comparativo mostrou que nos pediculados, a união do enxerto foi mais rápida. 21

## Tratamento Sistêmico

O osteossarcoma convencional era uma doença de tratamento exclusivamente cirúrgico antes do advento da quimioterapia. Apesar da estratégia proposta por Gross de amputação precoce, a sobrevida global em 5 anos ainda era de apenas 20%, 22 23 24 sendo que metástases pulmonares eram observadas após 6 a 12 meses, sugerindo a presença de micrometástases já ao diagnóstico.

A introdução da poliquimioterapia reduziu a disseminação sistêmica da doença e melhorou a sobrevida global em 2 anos em 50%, como demonstrado em por Eiber et al. 25 Em 1976, Rosen et al. 26 introduziram a quimioterapia neoadjuvante seguida de cirurgia conservadora que mostrou-se segura e permitiu a análise das margens cirúrgicas e da resposta histológica à quimioterapia. Entretanto, desde essa época a sobrevida não aumentou nos últimos 30 anos, apesar novas terapias têm sido continuamente pesquisadas.

Atualmente a terapia se baseia em esquemas de poliquimioterapia neoadjuvante, ressecção ampla e poliquimioterapia adjuvante. Os principais quimioterápicos são o metotrexato em altas doses, cisplatina e doxorrubicina. 27 Ifosfamida e etoposido também são agentes ativos e, geralmente utilizados em pacientes de alto risco e/ou recidivados.

A mudança das drogas baseada na resposta histológica à quimioterapia neoadjuvante foi avaliada e não conseguiu demonstrar melhora na sobrevida. O ensaio clínico *European and American Osteosarcoma Study Group* (EURAMOS-1) teve como objetivos primários determinar se a adição de ifosfamida e etoposido pós-operatória melhoraria os resultados nos casos de resposta ruim à quimioterapia neoadjuvante e se a adição de um tratamento de manutenção de 2 anos, com interferon alfa-2 melhoraria os resultados para bons respondedores. Infelizmente não houve diferença significativa entre os diferentes braços do estudo. 27

Aproximadamente 30% dos pacientes não metastáticos evoluem com recorrência, especialmente nos pulmões. A terapia de salvamento para esses pacientes ainda é pobre. A expectativa de sobrevida em 5 anos, é de apenas 25%. 28 Esquema de tratamento utilizando drogas como a gencitabina e docetaxel é uma opção terapêutica, mas com baixa resposta. A associação de temozolamida e etoposido já foi avaliada com potenciais benefícios, demonstrando ser uma possibilidade de terapia de resgate. 29

A quimioterapia metronômica é usada em baixas doses e a longo prazo atua nas células endoteliais dos neovasos formados no microambiente tumoral, inibindo a angiogênese local. Esse método apresentou resultados pouco encorajadores em pacientes não metastáticos. 30 Já para pacientes metastáticos, os resultados são mais favoráveis utilizando a ciclofosfamida, metotrexato e etoposido por via oral. Doença metastática já ao diagnóstico, a recidiva e a resistência aos múltiplos medicamentos (MDR) são os três maiores obstáculos para boa evolução clínica e melhores chances de cura.

O osteossarcoma é considerado um tumor relativamente radiorresistente, atualmente, a radioterapia é reservada para tumores irressecáveis e doença avançada, especialmente para controle de dor.

Os avanços recentes incluem a quimioterapia inalatória que visa permitir a exposição a altas doses de drogas direcionadas ao pulmão, importante sítio de metástases, e com baixa toxicidade sistêmica. 31 Essa modalidade de terapia pode, futuramente, permitir a utilização de imunoterapias como a interleucina 2 (IL-2). 32 Encontram-se na literatura alguns estudos pré-clínicos relacionados, particularmente o estudo em aberto, fase I /II com a utilização de gencitabina inalatória, que pode ser a primeira evidência de eficácia clínica, conduzido por Gordon et al. 33

A avaliação histológica e imunohistoquímica do tumor primário podem produzir marcadores prognósticos úteis e ajudar a programar um tratamento mais individualizado. A presença de invasão microscópica vascular, observada em alguns espécimes do tumor, pode estar associada a uma resposta pobre à quimioterapia neoadjuvante, a um alto risco de metástases e a uma menor sobrevida. Esta observação sugere que, nesses casos, poder-se-ia avaliar a realização de uma margem tumoral mais ampla bem como discutir a utilização de esquemas de tratamento adjuvante com agentes alternativos.

Sabe-se que o osteossarcoma é altamente heterogêneo, tanto na sua análise histológica quanto em sua classificação molecular. Nos últimos anos, vários estudos de sequenciamento de última geração revelaram importantes vias de sinalização no osteossarcoma. A terapia direcionada provavelmente será melhor sucedida quando o alvo correspondente estiver claro. Estudos tentam classificar os osteossarcomas com base em alterações genéticas frequentes e validadas. Em artigo de revisão publicado em 2021 por Chen et al., 34 o osteossarcoma foi classificado em 10 subtipos moleculares, dentre eles: osteossarcomas com amplificações da cíclica/CDK, MYC, MDM2, AURKB e RTK; amplificação de RTK; alteração de P13K/AKT; deleções de NF, BRCA, ATRX e mutação de IGF. Drogas das famílias dos inibidores de mTOR, MEK, EGF-1R, VEGFR, PARP estão em estudo ainda em fase pré-clínica.

Nos últimos anos, numerosos ensaios clínicos foram conduzidos com imunoterapias e receberam considerável atenção por sua eficácia no tratamento de vários tumores. Experimentos pré-clínicos de imunoterapias envolvendo a aplicação de vacinas tumorais, imunomoduladores, células T geneticamente modificadas, citocinas, inibidores de checkpoints imunológicos ou terapia combinada, estão em estudo para tratamento de osteossarcoma. Uma melhor compreensão da resposta imune ao osteossarcoma e o desenvolvimento de biomarcadores poderão aumentar o número de pacientes que se beneficiarão de imunoterapias. 35

A terapia com células CAR-T vem sendo estudada intensamente nos últimos anos, especialmente para tratamento de neoplasias hematológicas. Estudos utilizando a terapia para tratamento de tumores sólidos estão em andamento. Apesar de ainda de difícil acesso, pode ser promissora. Há um ensaio clínico em aberto para tratamento de sarcomas e neuroblastoma com anti GD2 positivo. 36 37

A sobrevida global em 5 anos atualmente é de cerca de 65% em estudos norte-americanos na década de 2000. Entretanto, desde então, os índices de sobrevida permanecem em um “ *plateau”* desde os estudos da década de 1970. Atualmente os fatores de pior prognósticos são metástase ao diagnóstico, sexo feminino, fosfatase alcalina aumentada, tumores secundários, expressão de microRNAs e índice de Huvos I e II ( Tabela 1 )

**Tabela 1:** Índice de necrose tumoral e resposta à quimioterapia neoadjuvante

RESPOSTA	ÍNDICE DE HUVOS	NECROSE
BOM RESPONDEDOR	IV	100%
BOM RESPONDEDOR	III	>90%
MAL RESPONDEDOR	II	50%-90%
MAL RESPONDEDOR	I	ATE 50%

## Considerações Finais

Avanços nas terapias sistêmica e de controle local seguem em evolução. Um melhor conhecimento da biologia e de seus mecanismos de disseminação são primordiais para uma abordagem terapêutica mais efetiva, especialmente para os pacientes de prognóstico mais desfavoráveis. A estratificação dos pacientes baseada em suas características genéticas e o desenvolvimento de terapias alvo são o futuro próximo.
